# Supplementary material for: Characterizing the mechanism of action for mRNA therapeutics for the treatment of propionic acidemia, methylmalonic acidemia, and phenylketonuria
Source: Nat Commun. 2024 May 7;15:3804. doi: 10.1038/s41467-024-47460-9 (PMC11076592; doi:10.1038/s41467-024-47460-9)
Supplement: Supplementary file 1 — Supplementary Information [file 41467_2024_47460_MOESM1_ESM.pdf]

Supplementary Information

Characterizing the Mechanism of Action for mRNA Therapeutics for the Treatment of Propionic Acidemia, Methylmalonic Acidemia, and Phenylketonuria

Baek et al

Supplementary Table 1. Summary of plasma hPCCA mRNA and hPCCB mRNA PK parameters in male and female *Pcca*$^{-/-}$ (A138T) mice and male and females combined following a single IV bolus injection of mRNA-3927.

**Analyte**	**hPCCA**			**hPCCB**		
Sex	Male (n=9)	Female (n=9)	Combined (n=18)	Male (n=9)	Female (n=9)	Combined (n=18)
Dose	1.0 mg/kg					
AUC_{tlast} (hr·ng/mL)	8600	6290	7400	7180	4720	5880
C_{max} (ng/mL)	1080	1080	1080	1010	776	892
$T_{1/2}$ (hr)	5.37	4.99	5.39	4.45	4.47	4.92
CL (mL/hr/kg)	116	159	135	139	212	170
V_z (mL/kg)	900	1140	1050	894	1370	1210
Dose	2.0 mg/kg					
AUC_{tlast} (hr·ng/mL)	29400	18800	24000	20700	9280	14900
C_{max} (ng/mL)	2530	1930	2180	1710	1080	1350
$T_{1/2}$ (hr)	6.13	4.07	5.50	5.72	4.40	5.47
CL (mL/hr/kg)	67.6	107	83.0	96.5	215	134
V_z (mL/kg)	598	626	659	797	1370	1060

AUC_{tlast}, area under the concentration versus time curve from the last start of dose administration to the time after dosing; C_{max}, maximum observed concentration; CL, clearance; hPCCA, human propionyl-CoA carboxylase α subunit; hPCCB, human propionyl-CoA carboxylase β subunit; IV, intravenous; mRNA, messenger RNA; PK, pharmacokinetic; $T_{1/2}$, terminal half-life; V_z, volume of distribution based on the terminal elimination phase.

Supplementary Table 2. Summary of plasma disease biomarker AUC below baseline in mRNA-3927 or control treated *Pcca*$^{-/-}$ (A138T) mice.

	**Control (Tris-sucrose) n=6**	**mRNA-3927 (0.5 mg/kg) n=5~6**
AUC_below_B (mean ± SD)		
2-MC (week·µM)	2.37 ± 2.32	8.71 ± 3.49
3-HP (week·µM)	37.7 ± 37.0	170 ± 163
C3/C2 ratio (week·ratio)	0.124 ± 0.187	1.23 ± 0.916
PCC Activity (mean ±SD)	2.72 ± 2.26	7.77 ± 4.27

2-MC; 2-methylcitrate; 3-HP, 3-hydroxypropionate; AUC_below_B; area under the curve that is below the baseline and above the response curve; C3/C2, ratio of propionylcarnitine to acetylcarnitine; mRNA, messenger ribonucleic acid; PCC, propionyl-CoA carboxylase; SD, standard deviation.

Supplementary Table 3. Summary of plasma hMUT mRNA PK parameters following a single IV bolus administration of mRNA-3705 in CD1 mice.

**Sex**	**Male n=12**	**Female n=12**	**Combined[a] n=24**
Dose[b]	0.1 mg/kg		
AUC_{tlast} (hr·ng/mL)	1340	662	1010
C_{max} (ng/mL)	517	232	375
$T_{1/2}$ (hr)	4.05	18.7[c]	6.42
CL (mL/hr/kg)	74.3	149[c]	99.2
V_z (mL/kg)	434	4020[c]	919
Dose	0.5 mg/kg		
AUC_{tlast} (hr·ng/mL)	13900	8290	11100
C_{max} (ng/mL)	3750	1480	2620
$T_{1/2}$ (hr)	4.90	4.43	4.93
CL (mL/hr/kg)	35.9	60.2	45.0
V_z (mL/kg)	253	385	320
Dose	1.0 mg/kg		
AUC_{tlast} (hr·ng/mL)	36600	26100	31200
C_{max} (ng/mL)	6140	4740	5150
$T_{1/2}$ (hr)	8.57	4.77	7.33
CL (mL/hr/kg)	27.1	38.2	31.8
V_z (mL/kg)	335	263	337

AUC_{tlast}, area under the concentration versus time curve from the last start of dose administration to the time after dosing; CL, clearance; C_{max}, maximum observed concentration; hMUT, human methylmalonyl-CoA mutase; PK, pharmacokinetic; IV, intravenous; mRNA, messenger RNA; $T_{1/2}$, terminal half-life; V_z, volume of distribution based on the terminal elimination phase.

[a]Sex differences were observed, values presented for information purpose only.

[b]Dose level refers to the average total amount of mRNA in the administered LNPs.

[c]Values presented for information purpose only.

Supplementary Table 4. Descriptive statistics of liver hMUT protein concentrations, liver MUT enzyme activity, and tissue methylmalonic acid concentrations in mRNA-3705 or control treated *Mut*$^{-/-}$;Tg$^{\text{INS-CBA-G715V}}$ mice at the end of the single-dose study.

	**Control n=4**	**mRNA-3705 (0.2 mg/kg) n=5**	**mRNA-3705 (0.5 mg/kg) n=4[a]**
Liver hMUT protein concentrations (ng/mg protein)			
Mean ± SD	0.0 ± 0.0	45.0 ± 42.2	70.4 ± 24.2
P value	-	0.0836	0.0136
Liver MUT enzyme activity (nmol/min/mg protein)			
Mean ± SD	2.8 ± 0.2	5.7 ± 2.8	10.7 ± 6.5
P-value	-	0.4577	0.0317
Plasma methylmalonic acid concentrations (µM)			
Baseline (Day -7)	892 ± 241	614 ± 190	589 ± 206
Day 1	1260 ± 280	425 ± 422	114 ± 20
P value	0.0732	0.4085	0.0201
Tissue methylmalonic acid concentrations 24 hours post-dose (nmol/g tissue)			
Liver			
Mean ± SD	4662.5 ± 1752.2	1091.9 ± 1457.5	7.9 ± 15.8
P value	-	0.0046	0.0011
Kidney			
Mean ± SD	3480.0 ± 1612.1	1094.0 ± 1245.6	189.7 ± 128.9
P value	-	0.0240	0.0052
Heart			
Mean ± SD	124.1 ± 37.1	43.6 ± 61.5	8.1 ± 16.3
P value	-	0.0411	0.0080

hMUT, human methylmalonyl-CoA mutase; mRNA, messenger RNA; SD, standard deviation.

[a]One animal in the mRNA-3705 mg/kg group received only a partial dose; therefore, the sample was removed from the analysis. For plasma methylmalonic acid, a two-way repeated measure ANOVA followed by Šídák's multiple comparisons test was used. For tissue methylmalonic acid concentrations, liver MUT protein concentrations, and liver protein activity, one-way ANOVAs followed by Dunnett’s pairwise comparison tests were performed. For all statistical tests, two-tailed p-values <0.05 were considered statistically significant.

Supplementary Table 5. Summary of serum mRNA from mRNA-3210 PK parameters in male or female PAH^{enu2} mice following a single IV dose of mRNA-3210.

**Sex**	**Male n=4**	**Female n=4**	**Combined n=8**
Dose	0.25 mg/kg		
AUC_{0-168} (hr·ng/mL)	2450	1780	2115
C_{max} (ng/mL)	453	434	444
Eff $T_{1/2}$ (hr)	4.89	3.46	4.18
CL (mL/hr/kg)	NA	141	-
V_z (mL/kg)	NA	3210	-
Dose	0.5 mg/kg		
AUC_{0-168} (hr·ng/mL)	5880	4620	5250
C_{max} (ng/mL)	876	959	918
Eff $T_{1/2}$ (hr)	4.80	3.64	4.22
CL (mL/hr/kg)	85.1	108	96.6
V_z (mL/kg)	2360	2560	2460
Dose	1.0 mg/kg		
AUC_{0-168} (hr·ng/mL)	11,200	31,700	21,450
C_{max} (ng/mL)	1640	1850	1750
Eff $T_{1/2}$ (hr)	5.29	19.9	12.6
CL (mL/hr/kg)	89.5	31.6	60.6
V_z (mL/kg)	2480	395	1438

AUC_{0-168}, area under the concentration-time curve from 0 to 168 hours; CL, clearance; C_{max}, maximum observed concentration; PK, pharmacokinetic; IV, intravenous; mRNA, messenger RNA; Eff $T_{1/2}$, Effective terminal half-life; V_z, volume of distribution based on the terminal elimination phase.

Supplementary Table 6. Summary of liver mRNA from mRNA-3210 PK parameters in male and female mice combined following a single IV dose of mRNA-3210

**PK parameter**	**mRNA-3210 (0.25 mg/kg) n=8**	**mRNA-3210 (0.5 mg/kg) n=8**	**mRNA-3210 (1.0 mg/kg) n=8**
AUC_{0-168} (hr·ng/mg/mg/kg)	1.03	1.96	6.28
C_{max} (ng/mg)	0.133	0.178	0.504
T_{max} (hr)	2	2	2
Eff $T_{1/2}$ (hr)	10.7	10.5	11.1

AUC_{0-168}, area under the concentration-time curve from 0 to 168 hours; C_{max}, maximum observed concentration; PK, pharmacokinetic; IV, intravenous; mRNA, messenger RNA; Eff $T_{1/2}$, Effective terminal half-life; T_{max}, time to maximum observed concentration.

Supplementary Table 7. Summary statistics for PD Parameters of liver hPAH protein and blood phenylalanine in from mRNA-3210 treated PAH[enu2] mice.

	**mRNA-3210 (0.25 mg/kg) n=8**	**mRNA-3210 (0.5 mg/kg) n=8**	**mRNA-3210 (1.0 mg/kg) n=8**
hPAH Protein[a]			
TE_{max} (hr)	24	24	8
E_{max} (ng/g)	6950	16,700	35,600
TE_{last} (h), median	96	168	168
$AUEC_{0-168}$ (h·ng/g), mean	498,000	1,130,000	2,090,000
Phenylalanine			
TE_{max} (h), median (min-max)	12 (1-24)	24 (24-24)	48 (12-48)
E_{max} (µmol/L), mean ± SD	1105 ± 209	561 ± 279	138 ± 78
TE_{last} (h), median (min-max)	168 (168-168)	168 (168-168)	168 (168-168)
AUC_below_B, mean ± SD	21,502 ± 15,533	64,960 ± 25,013	150,044 ± 52,699

AUC_{0-168}, area under the concentration-time curve from 0 to 168 hours; AUC_below_B; area under the curve that is below the baseline and above the response curve; C_{max}, maximum observed concentration; E_{max}, maximum effect; hPAH, human phenylalanine hydroxylase; mRNA, messenger ribonucleic acid; PD, pharmacodynamics; PK, pharmacokinetic; TE_{last}, time to last measurable effect; TE_{max}, time to maximum effect; T_{last}, time to last measurable concentration.

[a]PD parameters generated using sparse samples.

Supplementary Table 8. List of studies and analytes used for development of PK/PD the mRNA-3927 model.

Study	Test article	IV dose (mg/kg)	Dosing frequency	Species	Strain	Analytes
2308-060	mRNA-3927	1.0 and 2.0	Single dose	Mouse	*Pcca*$^{-/-}$ (A138T)	Plasma hPCCA and hPCCB mRNA
01070005	mRNA-3927	1.0, 3.0, and 9.0	Q2W × 3	Rat	Sprague-Dawley	Plasma hPCCA and hPCCB mRNA
5002694	mRNA-3927	1.0, 3.0, and 5.0	Q2W × 3	Monkey	Cynomolgus	Plasma hPCCA and hPCCB mRNA
2357, 2514, 2524	hPCCA/hPCCB mRNA[a]	1.0	Single dose	Mouse	*Pcca*$^{-/-}$ (A138T)	Liver hPCCA and hPCCB protein
3037	mRNA-3927	0.2, 0.5, 1.0, and 2.0	Single dose	Mouse	*Pcca*$^{-/-}$ (A138T)	Plasma 2-MC, 3-HP, and C3/C2
3082	mRNA-3927	0.5 and 2.0	Q3W × 4	Mouse	*Pcca*$^{-/-}$ (A138T)	Plasma 2-MC, 3-HP, and C3/C2

2-MC, 2-methylcitrate; 3-HP, 3-hydroxypropionate; C3/C2, ratio of propionylcarnitine to acetylcarnitine; hPCCA, human propionyl-CoA carboxylase α subunit; hPCCB, human propionyl-CoA carboxylase β subunit; IV, intravenous; mRNA, messenger RNA; PD, pharmacodynamic; PK, pharmacokinetic; Q2W, every 2 weeks; Q3W, every 3 weeks.

[a]PCCA and PCCB mRNA co-dosed in a 1:1 ratio.

Supplementary Table 9. List of studies and analytes used for development of PK/PD the mRNA-3705 model.

Study	Test article	IV dose (mg/kg)	Dosing frequency	Species	Strain	Analytes
20238405	mRNA-3705	0.1, 0.5, and 1.0	Single dose	Mouse	CD1	Plasma hMUT mRNA<br>Liver hMUT protein
	CX-020629 in SM86/DMG	1.0				
20238406	mRNA-3705	0.1, 0.5, and 1.0	Single dose	Rat	Sprague-Dawley	Plasma hMUT mRNA
2023408	mRNA-3705	0.2 and 1.0	Single dose	Monkey	Cynomolgus	Plasma hMUT mRNA
3767	mRNA-3705	0.2 and 0.5	QM × 2	Mouse	*Mut*$^{-/-}$;TgINS-CBA-G715V hypomorphic	Plasma methylmalonic acid
	CX-020629 in SM86/DMG	0.5				
3893	mRNA-3705	0.2, 0.5, and 1.0	6 doses administered on Day 0, Week 4, Week 6, Week 8, Week 10, and Week 12	Mouse	*Mut*$^{-/-}$;TgINS-CBA-G715V hypomorphic	Plasma methylmalonic acid
	CX-020629 in SM86/DMG	0.5				
		CX-020629 in				
3495	mRNA-3705	0.2 and 0.5	Single dose	Mouse	*Mut*$^{-/-}$;TgINS-CBA-G715V hypomorphic	Plasma methylmalonic acid

DMG, dimyristoyl glycerol; hMUT, human methylmalonyl-CoA mutase; IV, intravenous; LNP, lipid nanoparticle; mRNA, messenger RNA; PD, pharmacodynamic; PK, pharmacokinetic; QM, monthly.

Note: mRNA-3705 consists of CX-020629 mRNA formulated in SM-86/OL56 LNP; CX-020629 in SM-86/DMG consists of CX-020629 formulated in SM-86/DMG LNP.

Supplementary Table 10. List of studies and analytes used for development of PK/PD the mRNA-3210 model.

Study	Test article	IV dose (mg/kg)	Dosing frequency	Species	Strain	Analytes
5003937	mRNA-3210	0.25, 0.5, and 1.0	Single dose	Mouse	B6.BTRB-*Pah*^{enu2}	Blood Phe<br>Serum and liver hPAH mRNA<br>Liver hPAH protein
5003975	mRNA-3210	0.5	Single dose	Mouse	C57BL/6<br>B6.BTRB-*PAH*$^{enu2+/+}$ (wild-type)	Serum and liver hPAH mRNA<br>Liver hPAH protein
5003976	mRNA-3210	0.25, 0.5, and 1.0	QW × 3	Mouse	B6.BTRB-*PAH*^{enu2}	Blood Phe<br>Liver hPAH protein
20358989	mRNA-3210	0.5	Single dose	Monkey	Cynomolgus	Serum hPAH mRNA<br>Liver hPAH protein
20358990	mRNA-3210	1.0, 3.0, and 6.0	QW × 3	Monkey	Cynomolgus	Serum hPAH mRNA

enu2, N-ethyl-N-nitrosourea, hPAH, human phenylalanine hydroxylase; mRNA, messenger RNA; PAH, phenylalanine hydroxylase; PD, pharmacodynamic; Phe, phenylalanine; PK, pharmacokinetic; QW, weekly.
