# Supplementary material for: Characterizing the mechanism of action for mRNA therapeutics for the treatment of propionic acidemia, methylmalonic acidemia, and phenylketonuria
Source: Nat Commun. 2024 May 7;15:3804. doi: 10.1038/s41467-024-47460-9 (PMC11076592; doi:10.1038/s41467-024-47460-9)
Supplement: Supplementary file 3 — Reporting Summary [file 41467_2024_47460_MOESM3_ESM.pdf]

nature portfolio

Corresponding author(s): Patrick Finn, Lin Guey, and Paolo GV Martini

Last updated by author(s): Feb 1, 2024

Reporting Summary

Nature Portfolio wishes to improve the reproducibility of the work that we publish. This form provides structure for consistency and transparency in reporting. For further information on Nature Portfolio policies, see our Editorial Policies and the Editorial Policy Checklist.

Statistics

For all statistical analyses, confirm that the following items are present in the figure legend, table legend, main text, or Methods section.

n/a	Confirmed	
☐	☒	The exact sample size (*n*) for each experimental group/condition, given as a discrete number and unit of measurement
☐	☒	A statement on whether measurements were taken from distinct samples or whether the same sample was measured repeatedly
☐	☒	The statistical test(s) used AND whether they are one- or two-sided<br>*Only common tests should be described solely by name; describe more complex techniques in the Methods section.*
☒	☐	A description of all covariates tested
☐	☒	A description of any assumptions or corrections, such as tests of normality and adjustment for multiple comparisons
☐	☒	A full description of the statistical parameters including central tendency (e.g. means) or other basic estimates (e.g. regression coefficient) AND variation (e.g. standard deviation) or associated estimates of uncertainty (e.g. confidence intervals)
☐	☒	For null hypothesis testing, the test statistic (e.g. *F*, *t*, *r*) with confidence intervals, effect sizes, degrees of freedom and *P* value noted<br>*Give P values as exact values whenever suitable.*
☒	☐	For Bayesian analysis, information on the choice of priors and Markov chain Monte Carlo settings
☒	☐	For hierarchical and complex designs, identification of the appropriate level for tests and full reporting of outcomes
☒	☐	Estimates of effect sizes (e.g. Cohen's *d*, Pearson's *r*), indicating how they were calculated

Our web collection on statistics for biologists contains articles on many of the points above.

Software and code

Policy information about availability of computer code

Data collection	Data collection and assay methods for mRNA quantification and assessment of protein concentration, enzyme activity and metabolite concentration in the PA, MMA, and PKU PK and PD studies are described in the methods. PK/PD model evaluation was performed using appropriate diagnostics.<br>For the PA and MMA PK studies, mRNA from mRNA-3927 or mRNA-3705 was quantified by branched DNA analysis using the QuantiGene TM Singleplex assay kit according to the manufacturer's instructions (Thermo Fisher Scientific, Waltham, MA).<br>For the PKU study, mRNA from mRNA-3210 in both liver and serum samples were quantified by real-time reverse transcription-quantitative polymerase chain reaction (PCR) assay using the QuantStudio™ Real-Time PCR System (Thermo Fisher Scientific).<br>For the MMA study, protein quantitation was analyzed by liquid chromatography-tandem mass spectrometry (LC-MS/MS) analysis (Thermo Easy 1000 nano-UPLC, Orbitrap Fusion Mass Spectrometer; Thermo Fisher Scientific). For PKU, hPAH protein concentration was quantified by Extracts were analyzed using LC-MS/MS in positive ionization mode under optimized conditions for detection of LFE-positive ions formed by electrospray ionization.
Data analysis	In the PA PK study, statistics were generated using Phoenix, version 8.1. In the PA PD study, statistical analyses were performed using GraphPad Prism, version 7.01. In the MMA PK study, statistical analyses were performed using Phoenix, version 1.4. For the MMA PD study, statistical analyses were performed using GraphPad Prism, version 9. For the PKU PK/PD study, statistical analyses were performed using Phoenix, version 8.3. In the modeling study, data pre- and post-processing were performed using RStudio (version 4 and higher).

For manuscripts utilizing custom algorithms or software that are central to the research but not yet described in published literature, software must be made available to editors and reviewers. We strongly encourage code deposition in a community repository (e.g. GitHub). See the Nature Portfolio guidelines for submitting code & software for further information.

Data

Policy information about availability of data

All manuscripts must include a data availability statement. This statement should provide the following information, where applicable:

- Accession codes, unique identifiers, or web links for publicly available datasets
- A description of any restrictions on data availability
- For clinical datasets or third party data, please ensure that the statement adheres to our policy

The datasets generated during and/or analyzed during the current study are not publicly available due to the propriety nature of the LNP therapeutics described herein. Access to data and supporting documents from qualified external researchers may be available upon request. Source data for presented figures are provided with this paper.

Research involving human participants, their data, or biological material

Policy information about studies with human participants or human data. See also policy information about sex, gender (identity/presentation), and sexual orientation and race, ethnicity and racism.

Reporting on sex and gender	NA
Reporting on race, ethnicity, or other socially relevant groupings	NA
Population characteristics	NA
Recruitment	NA
Ethics oversight	NA

Note that full information on the approval of the study protocol must also be provided in the manuscript.

Field-specific reporting

Please select the one below that is the best fit for your research. If you are not sure, read the appropriate sections before making your selection.

☒ Life sciences ☐ Behavioural & social sciences ☐ Ecological, evolutionary & environmental sciences

For a reference copy of the document with all sections, see nature.com/documents/nr-reporting-summary-flat.pdf

Life sciences study design

All studies must disclose on these points even when the disclosure is negative.

Sample size	We have used at least three replicates for each experiment unless stated otherwise. Sample sizes were selected based on historical experience with these animal models and endpoints analyzed
Data exclusions	No data was excluded.
Replication	The number of replicates is indicated in the figure legends.
Randomization	This was not a randomized study. Age of animals included in the studies are provided in the Methods section. When an age range is provided, animals were randomized across groups to ensure equal distribution of age across groups.
Blinding	Experiments were not blinded. However samples were blinded to the analysts during sample analysis.

Reporting for specific materials, systems and methods

We require information from authors about some types of materials, experimental systems and methods used in many studies. Here, indicate whether each material, system or method listed is relevant to your study. If you are not sure if a list item applies to your research, read the appropriate section before selecting a response.

Materials & experimental systems

n/a	Involved in the study
☒	☐ Antibodies
☒	☐ Eukaryotic cell lines
☒	☐ Palaeontology and archaeology
☐	☒ Animals and other organisms
☒	☐ Clinical data
☒	☐ Dual use research of concern
☒	☐ Plants

Methods

n/a	Involved in the study
☒	☐ ChIP-seq
☒	☐ Flow cytometry
☒	☐ MRI-based neuroimaging

Animals and other research organisms

Policy information about studies involving animals; ARRIVE guidelines recommended for reporting animal research, and Sex and Gender in Research

Laboratory animals	For the PA PK study, Pcca–/– (A138T) mice (Jackson Laboratories, Bar Harbor, ME) aged 5.5 to 6 months were used. For the PA PD study, Pcca–/– (A138T) mice (Jackson Laboratories) aged 8 to 10 weeks were used. In the MMA PK study, CD1 (ICR) mice (Charles River Kingston, New York, NY) at least 8 weeks of age were used. In the MMA PD study, groups of Mut-/-;TgINS-CBA-G715V mice aged 5 to 11 weeks were used. For the PKU PK/PD study, PAHenu2 mice aged 11 to 17 weeks (Jackson Laboratories) were used. Target temperatures of 68 Fahrenheit (F) to 79F with a relative target humidity of 30% to 70% were maintained. A 12-hour light/12-hour dark cycle was maintained.
Wild animals	Wild type animals were included in the PCC protein activity analysis for mRNA-3927 (see Figure 2)
Reporting on sex	Both male and female mice were used to assess pharmacokinetics/pharmacodynamics (PD/PD) in all murine models. For PK analyses, mRNA concentrations derived from mRNA-3927, mRNA-3705, or mRNA-3210 were described in both male and female mice in murine models of propionic acidemia (PA), methylmalonic acidemia (MMA), and phenylketonuria (PKU), respectively.
Field-collected samples	No field-collected samples were used in the studies.
Ethics oversight	All animal experiments involving Pcca–/–(A138T) or Mut-/-;TgINS-CBA-G715V mice were approved and conducted in accordance with the regulations of the ModernaTX, Inc. Institutional Animal Care and Use Committee (IACUC). Protocols and amendments or procedures involving CD1 mice were reviewed and approved by Charles Rivers Laboratories IACUC (Shrewsbury, MA). Study plans, amendments, and procedures involving PAHenu2 mice were reviewed and approved by Charles River Laboratories IACUC (Montreal ULC, Sherbrooke Site). During the study, the care and use of PAHenu2 mice was conducted with guidance from the USA National Research Council and the Canadian Council on Animal Care (CCAC).

Note that full information on the approval of the study protocol must also be provided in the manuscript.
